# Seasonal changes in pollen limitation and femaleness along the snowmelt gradient in a distylous alpine herb, *Primula modesta*

**DOI:** 10.1002/ece3.1803

**Published:** 2015-10-30

**Authors:** Yoshiaki Kameyama, Manami Watanabe, Hideki Kurosawa, Takuya Nishimori, Daisuke Matsue, Masaaki Takyu

**Affiliations:** ^1^ Faculty of Regional Environment Science Tokyo University of Agriculture Tokyo 156‐8502 Japan; ^2^ Present address: OTA Floriculture Auction Co., Ltd. Tokyo 143‐0001 Japan; ^3^ Present address: Tamamura‐machi Sawa‐gun Gunma 370‐1105 Japan; ^4^ Present address: Hakone Botanical Garden of Wetlands Kanagawa 205‐0631 Japan

**Keywords:** Alpine ecosystem, asymmetrical disassortative pollination, flowering phenology, functional gender, heterostyly, pollinator availability, reproductive success

## Abstract

Flowering phenology of alpine plants is strongly determined by the timing of snowmelt, and the conditions of pollination of widely distributed plants vary greatly during their flowering season. We examined the reproductive success of the distylous alpine herb, *Primula modesta*, along the snowmelt gradient under natural conditions, and compared it with the result of artificial pollination experiments. In addition, the compositions and visit frequencies of pollinators to the flower of *P. modesta* were examined during the flowering period. The pin and thrum plants of *P. modesta* growing at the same site have an equal ability to produce seeds if a sufficient amount of legitimate pollen grains are deposited on the stigma surface. However, under natural conditions, their seed‐set success was often (even if not always) restricted by pollen limitation, and the functional gender of the pin and thrum plants biased to the female and male, respectively, associated with their growing sites. These variations were not ascribed to resource limitation nor biased morph ratio but to the seasonal changes in pollination situations, a replacement of pollinator types from long‐ to short‐tongued pollinators resulted in unidirectional pollen transfer from long stamens (thrum plants) to long styles (pin plants). The functional gender specialization may enhance the evolution of dioecy from heterostyly, but the severe pollen limitation may cause the breakdown of heterostyly into homostyly. To consider the evolutionary pathway of heterostylous plants, an accumulation of the empirical data is required demonstrating how phenological synchrony between plants and pollinators is decided and to what degree this relationship is stable over years, along with estimates of selection and gene flow in individual plants.

## Introduction

Seed production by plants is often restricted by the quantity and quality of pollen grains deposited on the stigma surface (Burd 1994; Larson and Barrett 2000; Ashman et al. 2004; Knight et al. 2005; Aizen and Harder 2007; Harder and Aizen 2010; Alonso et al. 2013; Wolowski et al. 2013; Arceo‐Gómez and Ashman 2014). This effect, termed pollen limitation, has been studied by both ecological and evolutionary biologist from the several viewpoints: for example, population dynamics, life histories, floral traits, and reproductive strategies in plants. However, the causes and consequences of pollen limitation are still unclear, because they vary greatly depending on pollination situations (e.g., pollinator composition and activity), mating system (e.g., self‐compatibility), and genetic load (e.g., inbreeding depression) of individual plant (Kameyama and Kudo 2015).

Heterostyly is a mating system in which anthers and stigmas are placed reciprocally between the two (distyly) or three (tristyly) types of floral morphs (Darwin 1877; Barrett 2002; Barrett and Shore 2008). Because most heterostylous species possess a heteromorphic self‐incompatibility system that limits or prevents self‐ and intramorph mating (Barrett and Shore 2008), reproductive success of heterostylous species strongly depends on pollen transfer between different morphs, termed legitimate pollination (Darwin 1877). As reported by Barrett (2002), heterostyly is a mating strategy that simultaneously reduces selfing and sexual interference, while promoting efficient cross‐pollination between morphs, a process known as disassortative pollination (Darwin 1877; Lloyd and Webb 1992; Barrett 2002; Lau and Bosque 2003; Barrett and Shore 2008).

The disassortative pollination hypothesis of Darwin (1877) has been examined by many studies (Ganders 1974, 1979; Barrett and Glover 1985; Barrett and Wolfe 1986; Wolfe and Barrett 1989; Stone 1995; Ree 1997; Nishihiro and Washitani 1998; Nishihiro et al. 2000; Ornelas et al. 2004; Sánchez et al. 2010; Valois‐Cuesta et al. 2011; Keller et al. 2014; Simón‐Porcar et al. 2014), and the results generally support the Darwin's hypothesis (1877); the proportion of legitimate pollen grains deposited on the stigmas of each morph is greater than expected from random pollination (Barrett and Shore 2008). However, most of these studies examined the pollen transfer by “long‐tongued” legitimate pollinators, which probe for nectar at the bottom of the floral tube and transfer legitimate pollen grains between reciprocal morphs. This means that changes in pollination situations may disrupt the disassortative pollination system in heterostylous species.

For example, Beach and Bawa (1980) proposed that short‐tongued pollinators may cause unidirectional pollen transfer from long stamens to long styles, resulting in asymmetrical disassortative pollination; functional gender of the pin and thrum plants becomes to more female biased and male biased, respectively. On the other hand, several studies suggested that insufficient pollen transfer by illegitimate pollinators may cause significant pollen limitation, which may be associated with the repeated breakdown of heterostyly into homostyly: a mating system where the anthers and stigmas are placed at the same height in accordance with the loss of self‐incompatibility (Barrett 1988, 1989; Washitani et al. 1994; Guggisberg et al. 2006; Sakai and Wright 2008; Haddadchi and Fatemi 2015). However, surprisingly few studies have examined the relationship between pollen limitation and functional gender (femaleness) of heterostylous plants.

Alpine ecosystem can be an excellent experimental system to consider seasonal changes in plant–pollinator interactions. Alpine plants usually bloom soon after snowmelt, and the length of flowering season is restricted to a short period (Holway and Ward 1965; Kudo 1991; Kudo and Suzuki 1999; Larl and Wagner 2006; Lambert et al. 2010). However, the timing of snowmelt greatly varies because of the heterogeneity of local topographical features (Thomson 2010). Thus, some plant species widely distributed along the snowmelt gradient continues to bloom over a month as the season progresses (Kudo 1991, 1996). In addition, the composition and activity of pollinators vary considerably during the season and affect the reproductive success of alpine plants (Kudo and Suzuki 2002; Kasagi and Kudo 2003, 2005; Hirao et al. 2006; Kudo 2006, 2014; Kameyama and Kudo 2009, 2015; Forrest and Thomson 2011; Forrest et al. 2011; Kudo et al. 2011). In addition, a delay of snowmelt may restrict photosynthetic carbon gain due to a very short growth period, resulting in a resource limitation (Kudo 1991; Berdanier and Klein 2011; Mallik et al. 2011; also see Leffler and Welker 2013). The reproductive success of alpine plants is apparently determined by both pollen and resource limitation.

**Figure 1:**
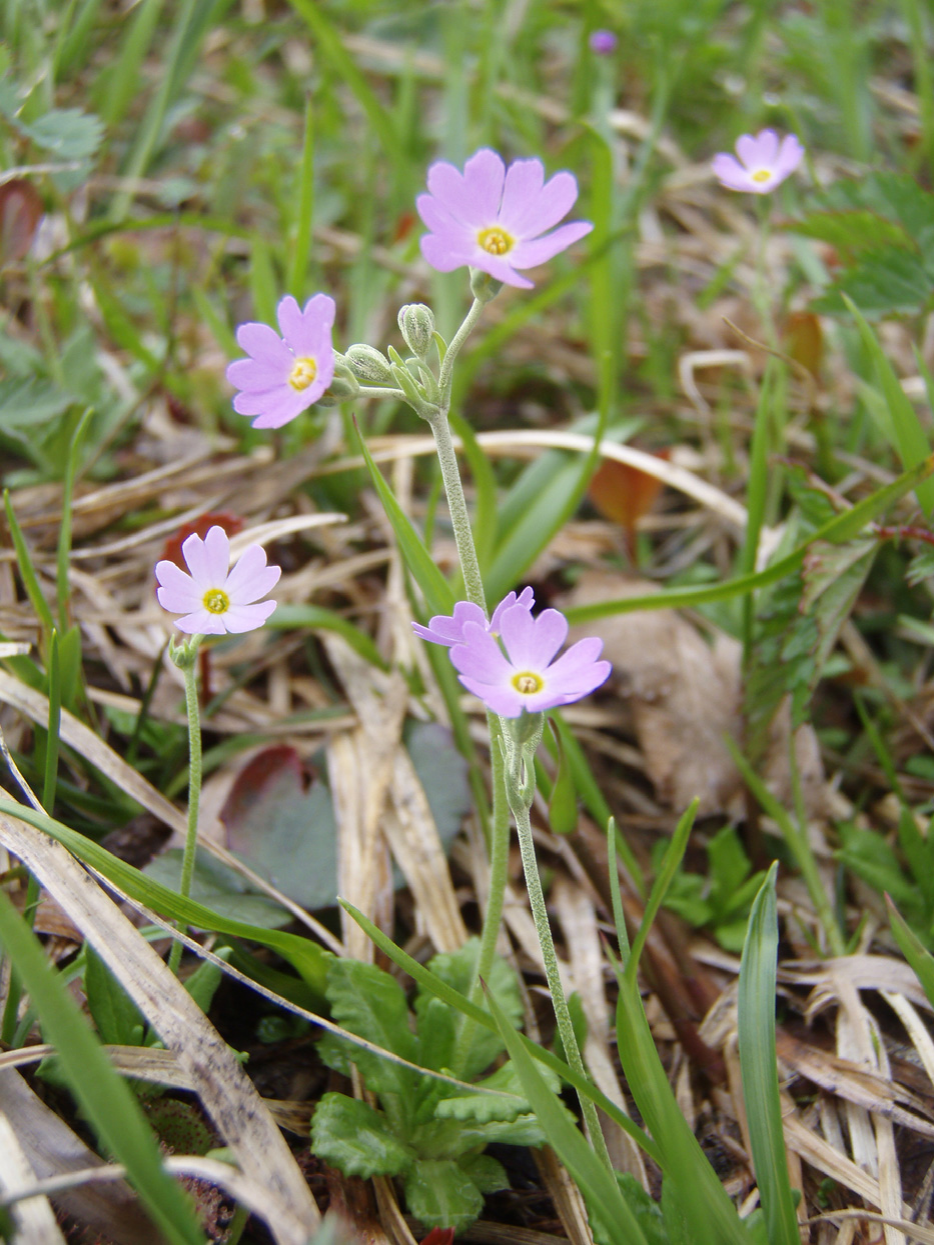
*Primula modesta* Bisset et Moore (Primulaceae) is a distylous perennial herb, distributed over a wide range of montane to alpine habitats throughout Japan.

In the current study, we examine the reproductive success of distylous alpine herb, *Primula modesta* Bisset et Moore (Fig. 1), along the snowmelt gradient. We expect that (1) if resource limitation is essential to determine the reproductive success of *P. modesta*, the potential ability to produce seeds, such as the number of flowers per plant, ovules per fruit, and seed to ovule ratio by experimental crosses, is smaller at the later snowmelt sites, (2) if pollen limitation is severe, the number of seeds produced under natural conditions is generally smaller than that produced by experimental crosses, and (3) if illegitimate pollinators cause unidirectional pollen transfer from long stamens to long styles, functional gender of the pin and thrum plants becomes to more female biased and male biased, respectively.

## Materials and Methods

### Study species and site

*Primula modesta* Bisset et Moore (Primulaceae) is a distylous perennial herb, distributed over a wide range of montane to alpine habitats throughout Japan (Fig. 1). This species produces one flowering stem with two to four flowers and possesses a heteromorphic self‐incompatibility system that almost completely prevents self‐ and intramorph mating (Wedderburn and Richards 1990; Shimono and Washitani 2007).

**Figure 2:**
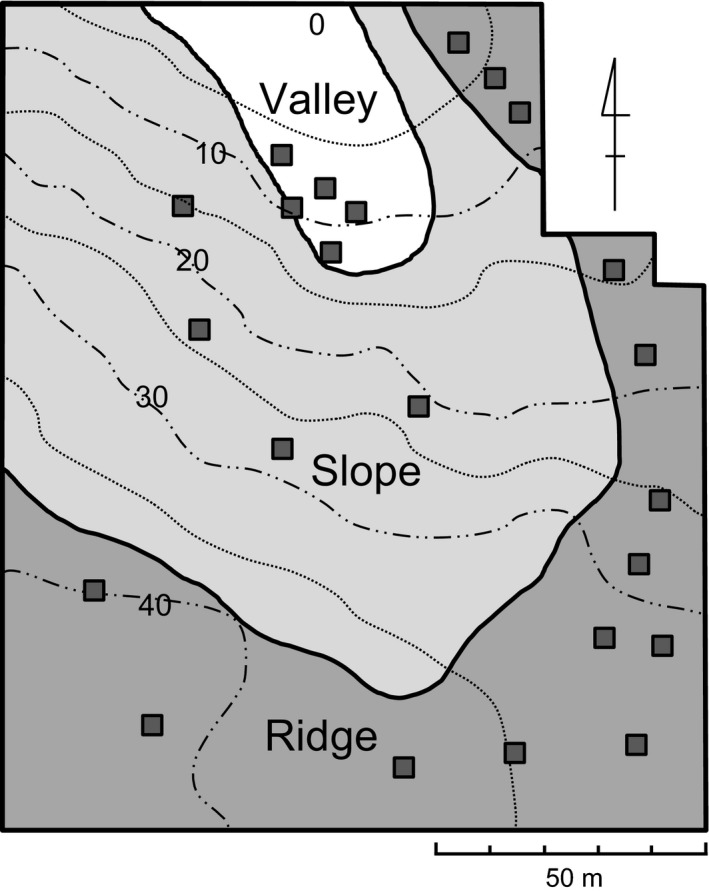
Map of the study area (130* *×* *150* *m). The contour lines show the relative height from the bottom of the valley at 5‐m intervals. Snow cover disappears during early April, mid‐June, and early July within the ridges, slopes, and valleys, respectively. Flowering of *Primula modesta* progresses from ridge to valley along the snowmelt gradient, with the peak flowering occurring during early June, early July, and late July at the ridges, slopes, and valleys, respectively. We set 14, 4, and 5 quadrats (0.5* *×* *0.5 m) at the ridges, slopes, and valleys, respectively (square symbol). Studies of *Primula modesta* were conducted within and nearby these quadrats, while pollinator observations were conducted within areas of 2 × 2 m during peak flowering at each site.

The current study was conducted within an area of 130 × 150 m at the north‐facing gentle slope of the Happo‐One ridge, northern Japan Alps, central Japan (N36°42′0″, E137°50′58″) (Fig. 2). At the study area (2000 m a.s.l.), annual mean temperature is 3.0°C, and the minimum and maximum daily mean temperature are −17.7°C and 25.6°C, respectively (data from November 2010 to October 2011). At the Hakuba village situated at the foot of a study area (703 m a.s.l.), mean annual precipitation and maximum snow depth are 2097 and 970 mm, respectively (data from 1981 to 2010). Snowmelt at the study area progresses from ridge to valley due to the differences in snow depth. The snow cover disappears during early April, mid‐June, and early July within the ridges, slopes, and valleys, respectively. Flowering of *P*. *modesta* progresses from ridges to valleys, with the peak flowering occurring during early June, early July, and late July at the ridges, slopes, and valleys, respectively.

### Plant reproduction

We set 14, 4, and 5 quadrats of the size 0.5 × 0.5 m at the ridges, slopes, and valleys, respectively (Fig. 2). During 2009 and 2010, we counted the number of plants, flowering plants, flowers per plant, and total number of flowers within each quadrat, discriminating between morphs (pin and thrum). To estimate the number of ovules and the seeds per fruit under natural conditions at each site (ridge, slope, and valley), we examined the plants growing within and nearby the quadrats over 2 years (2010 and 2011). The total number of pin and thrum plants examined was 78 and 79 during 2010 and 170 and 157 during 2011, respectively. We collected several fruits from each plant (2.8 on average) during 2010, and one fruit per plant during 2011. The total number of fruits collected from the pin and thrum plants was 233 and 204 during 2010 and 170 and 157 during 2011, respectively. The fruits were collected just before dehiscence, and the number of seeds and undeveloped seeds or ovules were counted using a microscope, and the seed‐set was calculated as follows: the number of ovules is the estimates of (the number of seeds) + (the number of undeveloped seeds or ovules), and the seed to ovule ratio is the estimates of (the number of seeds)/(the number of ovules).

To estimate the potential ability to produce seeds at each site (ridge, slope, and valley), we conducted artificial pollination experiments within and nearby the quadrats over 2 years (2010 and 2011). The total numbers of pin and thrum plants pollinated were 78 and 77 during 2010 and 59 and 81 during 2011, respectively. The plants were covered with fine‐meshed nylon nets to exclude insect visitors. For each plant, one target flower bud was emasculated just before opening, and other flowers were removed. After opening of the target flowers, hand pollination was conducted by legitimate pollen grains collected from a single pollen donor growing at least 5 m away from the recipient plant. All fruits derived from the pollination treatments were harvested just before dehiscence, and the number of seeds per fruit and the seed to ovule ratio was estimated as described above.

Using the data obtained, we estimated the pollen limitation of the pin and thrum plants at each site (ridge, slope, and valley) over 2 years (2010 and 2011) as follows: 1 − (the mean number of seeds per fruit produced under natural conditions)/(the mean number of seeds per fruit produced by artificial pollination treatments). In addition, we estimated the average femaleness of the pin and thrum plants as follows: (the mean number of seeds produced by the target morph)/[(the mean number of seeds produced by the target morph) + (the mean number of seeds produced by the other morph)]. This formula is appropriate for the self‐ and intramorph incompatible plants such as *P*. *modesta* (Wedderburn and Richards 1990; Shimono and Washitani 2007) under the 1:1 morph ratio, because under this situation, the average femaleness of one morph is exactly equal to the average maleness of the other morph (Lloyd 1979).

### Pollinator visits

We observed pollinator visits to the flowers of *P. modesta* during peak flowering at each site: early June, early July, and late July at the ridge, slope, and valley sites, respectively. Field observations were conducted during calm and fine periods during daylight hours (9:00 am–3:00 pm). The total observation times were 25 h per 5 days, 12 h per 3 days, and 14 h per 4 days, at the ridge, slope, and valley sites, respectively. On each observation day, we arbitrary set 2 × 2 m plot and recorded the pollinators and the number of flowers of *P. modesta*. Pollinators were classified into Diptera, Hymenoptera, Lepidoptera, and others during field observations, and major pollinators were collected and identified at the family or species level if possible.

### Statistical analyses

The effects of year (2009 and 2010), site (ridge, slope, and valley site), morph (pin and thrum), and the interaction of site and morph on the number of (a) plants, (b) flowering plants, (c) flowers per plant, and (d) flowers in total within quadrats were estimated by the generalized linear mixed‐effect model (GLMM), where quadrats were treated as a random factor, and a Poisson distribution with a log‐link function was postulated. The chi‐square‐ test was conducted to examine the deviation from a 1:1 ratio for the number of pin and thrum plants and their flowers in total. Here the data obtained at each quadrat were combined for each site, and the statistical significance was estimated after Bonferroni correction for multiple comparisons over sites (ridge, slope, and valley sites) and years (2009 and 2010).

The effects of year (2010 and 2011), site (ridge, slope, and valley sites), morph (pin and thrum), and the interaction of site and morph on (a) the number of ovules per fruit, (b) the seed to ovule ratio by experimental crosses, and (c) the seed to ovule ratio under natural conditions were estimated by the GLMM. In the analysis of (a), plants were treated as a random factor and a Poisson distribution with a log‐link function was postulated. In the analyses of (b) and (c), fruits were treated as a random factor and a binomial distribution with a logit‐link function was postulated. The GLMMs were conducted using R version 2.11.1 (R Development Core Team 2010), and the model selection based on Akaike's information criteria (AIC) was performed.

## Results

### Plant reproduction

The number of plants, flowering plants, flowers per plant, and flowers in total within the 0.5 × 0.5 m quadrats is shown in Table 1a–d. No effect of year and site on the number of plants was evident (Table 2a); however, significant yearly variations in the number of flowering plants, flowers per plant, and flowers in total were observed (Table 2b−d). The number of flowers per plant was significantly larger at the ridge with no relation to their morphs (Table 2c), and the number of flowering plants and the number of flowers in total were significantly larger for thrum morphs growing at the ridge (Table 2b and d). No deviation from the ratio of 1:1 was observed at each site (ridge, slope, and valley sites) in terms of the number of pin and thrum plants (chi‐squared‐test, adjusted *P* > 0.05), whereas significant deviations in the pin and thrum flowers were detected at the ridge during 2010 (chi‐squared‐test, adjusted *P* = 0.042) and at the valley during 2009 (chi‐squared‐test, adjusted *P* = 0.021), respectively.

**Table 1:** Number of (a) plants, (b) flowering plants, (c) flowers per plant, and (d) flowers in total within 0.5 × 0.5* *m quadrats across 2 years (2009 and 2010), three sites (ridge, slope, and valley), and two morphs (pin and thrum) in *Primula modesta*. Number of quadrats was 14, 4, and 5 at the ridge, slope, and valley, respectively. Data shown are mean* *±* *1 standard deviation

	2009		2010	
(a) *N* of plants				
Ridge	22.4 ± 7.8		23.1 ± 11.3	
Slope	28.3 ± 4.0		21.5 ± 7.3	
Valley	25.2 ± 7.5		26.4 ± 11.3	
	Pin	Thrum	Pin	Thrum
(b) *N* of flowering plants				
Ridge	5.0 ± 2.9	5.8 ± 3.5	3.4 ± 2.1	4.9 ± 3.2
Slope	5.8 ± 1.9	4.5 ± 2.4	4.3 ± 2.2	2.8 ± 2.5
Valley	4.0 ± 2.8	3.0 ± 1.9	3.8 ± 1.6	4.2 ± 2.3
(c) *N* of flowers per plant				
Ridge	4.1 ± 1.7	4.2 ± 1.9	3.7 ± 1.8	3.4 ± 1.5
Slope	3.0 ± 1.8	2.7 ± 1.1	2.3 ± 1.0	3.2 ± 0.9
Valley	3.2 ± 1.6	2.3 ± 0.7	2.8 ± 1.3	2.3 ± 1.0
(d) Total *N* of flowers				
Ridge	20.6 ± 11.1	24.4 ± 15.1	12.4 ± 8.4	16.3 ± 12.8
Slope	17.5 ± 9.5	12.3 ± 7.8	8.8 ± 4.3	8.8 ± 8.3
Valley	12.8 ± 12.5	7.0 ± 4.3	10.8 ± 6.0	9.6 ± 5.0

**Table 2:** The effects of year (2009 and 2010), site (ridge, slope, and valley), morph (pin and thrum), and the interaction of site and morph on the number of (a) plants, (b) flowering plants, (c) flowers per plant, and (d) flowers in total estimated by GLMM. Best‐fit model by AIC is shown

	Coefficient	SE	*Z* value	*P* value
(a) *N* of plants (df = 43, AIC = 90.4)^a^				
Intercept (2009, slope)	3.122	0.0861	36.248	<0.001
Year (2010)	−0.022	0.0605	–0.363	0.717
(b) *N* of flowering plants (df = 84, AIC = 135.1)				
Intercept (2009, slope, pin)	1.647	0.248	6.645	<0.001
Year (2010)	−0.216	0.099	−2.169	0.030
Ridge	−0.189	0.278	−0.678	0.498
Valley	−0.218	0.334	−0.653	0.514
Morph (thrum)	−0.322	0.244	−1.319	0.187
Ridge × Morph (thrum)	0.563	0.273	2.061	0.039
Valley × Morph (thrum)	0.242	0.336	0.719	0.472
(c) *N* of flowers per plant (df = 403, AIC = 280.0)				
Intercept (2009, slope, pin)	1.051	0.114	9.235	<0.001
Year (2010)	−0.139	0.055	−2.548	0.011
Ridge	0.372	0.125	2.982	0.003
Valley	−0.009	0.151	−0.059	0.953
(d) Total *N* of flowers (df = 84, AIC = 386.1)				
Intercept (2009, slope, pin)	2.587	0.259	9.976	<0.001
Year (2010)	−0.389	0.054	−7.197	<0.001
Ridge	0.293	0.291	1.008	0.314
Valley	0.000	0.346	0.000	1.000
Morph (thrum)	−0.223	0.146	−1.524	0.127
Ridge × Morph (thrum)	0.432	0.159	2.710	0.007
Valley × Morph (thrum)	−0.129	0.205	−0.628	0.530

AIC, Akaike's information criteria; GLMM, generalized linear mixed‐effect model.

a The dependent variables used here are year and site.

The number of ovules per fruit, seeds per fruit produced by experimental crosses, and seeds per fruit produced under natural conditions is shown in Table 3a–c. The number of ovules per fruit was significantly larger during 2011 and decreased from the ridge to the valley, with intermediate values evident at the slope (Table 4a). The seed to ovule ratio by experimental crosses was significantly lower during 2011 and higher at the ridge with no difference between the slope and the valley (Table 4b). No effect of the morph (pin and thrum) and interaction between the morph and site was observed for the number of ovules per fruit and seed to ovule ratio by experimental crosses. In contrast, the seed to ovule ratio under natural conditions was highest at the ridge, lowest at the slope, and intermediate at the valley, where the thrum plants showed a lower seed to ovule ratio associated with their growing site: The differences between morphs were largest at the slope, smallest at the ridge, and intermediate at the valley site (Table 4c).

**Table 3:** Number of (a) ovules per fruit, (b) seeds per fruit produced by experimental crosses, and (c) seeds per fruit produced under natural conditions across 2 years (2010 and 2011), three sites (ridge, slope, and valley), and two morphs (pin and thrum) in *Primula modesta*. Values are mean* *±* *1 standard deviation, and the number of samples is indicated in parentheses. Pollen limitation is the estimates of 1* *−* *(c)/(b). Femaleness is the estimates of [(c) of the target morph]/[(c) of the target morph + (c) of the other morph], assuming the 1:1 morph ratio and no illegitimate fertilization (Lloyd 1979)

	2010		2011	
	Pin	Thrum	Pin	Thrum
(a) *N* of ovules per fruit				
Ridge	56.6 ± 6.9 (70)	54.8 ± 8.7 (48)	57.4 ± 5.3 (68)	61.0 ± 4.8 (61)
Slope	53.9 ± 5.0 (87)	53.6 ± 4.5 (74)	56.0 ± 4.7 (49)	53.5 ± 4.6 (55)
Valley	40.9 ± 7.9 (76)	39.8 ± 7.6 (82)	50.4 ± 2.2 (53)	51.9 ± 3.2 (41)
(b) *N* of seeds per fruit produced by experimental crosses				
Ridge	50.5 ± 9.9 (40)	51.3 ± 9.3 (39)	33.3 ± 12.9 (41)	31.5 ± 12.1 (43)
Slope	40.1 ± 8.5 (19)	44.1 ± 7.1 (19)	29.5 ± 15.1 (10)	23.7 ± 16.2 (30)
Valley	36.3 ± 7.4 (19)	31.8 ± 7.4 (19)	11.4 ± 5.0 (8)	24.3 ± 11.7 (8)
(c) *N* of seeds per fruit produced under natural conditions				
Ridge	27.0 ± 18.1 (70)	26.4 ± 21.1 (48)	31.9 ± 13.5 (68)	34.0 ± 18.4 (61)
Slope	10.5 ± 12.8 (87)	6.3 ± 11.7 (74)	6.2 ± 12.0 (49)	2.3 ± 6.2 (55)
Valley	17.7 ± 9.9 (76)	19.6 ± 13.2 (82)	10.7 ± 11.1 (53)	4.2 ± 8.4 (41)
(d) Pollen limitation				
Ridge	0.47	0.49	0.04	−0.08
Slope	0.74	0.86	0.79	0.90
Valley	0.51	0.38	0.06	0.83
(e) Femaleness				
Ridge	0.51	0.49	0.48	0.52
Slope	0.63	0.38	0.73	0.27
Valley	0.47	0.53	0.72	0.28

**Table 4:** The effects of year (2010 and 2011), site (ridge, slope, and valley), morph (pin and thrum), and the interaction of site and morph on (a) the number of ovules per fruit, (b) the seed to ovule ratio by experimental crosses, and (c) the seed to ovule ratio under natural conditions estimated by GLMM. Best‐fit model by AIC is shown

	Coefficient	SE	*Z* value	*P* value
(a) *N* of ovules per fruit (df = 758, AIC = 638.4)				
Intercept (2010, slope, pin)	3.952	0.012	343.50	<0.001
Year (2011)	0.095	0.011	8.780	<0.001
Ridge	0.030	0.013	2.340	0.019
Valley	−0.188	0.014	−13.87	<0.001
Morph (thrum)	−0.004	0.011	−0.39	0.693
(b) Seed to ovule ratio by experimental crosses (df = 290, AIC = 891.6)				
Intercept (2010, slope, pin)	1.056	0.108	9.830	<0.001
Year (2011)	−1.430	0.099	−14.40	<0.001
Ridge	0.535	0.115	4.645	<0.001
Valley	−0.116	0.151	−0.770	0.442
(c) Seed to ovule ratio under natural conditions (df = 756, AIC = 3009)				
Intercept (2010, slope, pin)	−3.128	0.2548	−12.275	<0.001
Year (2011)	−1.221	0.203	−6.016	<0.001
Ridge	3.594	0.329	10.937	<0.001
Valley	2.307	0.335	6.890	<0.001
Morph (thrum)	−1.812	0.372	−4.867	<0.001
Ridge × Morph (thrum)	1.766	0.494	3.577	<0.001
Valley × Morph (thrum)	1.396	0.498	2.802	0.005

AIC, Akaike's information criteria; GLMM, generalized linear mixed‐effect model.

Number of ovules: (the number of seeds) + (the number of undeveloped seeds or ovules). Seed to ovule ratio: (the number of seeds)/(the number of ovules).

**Figure 3:**
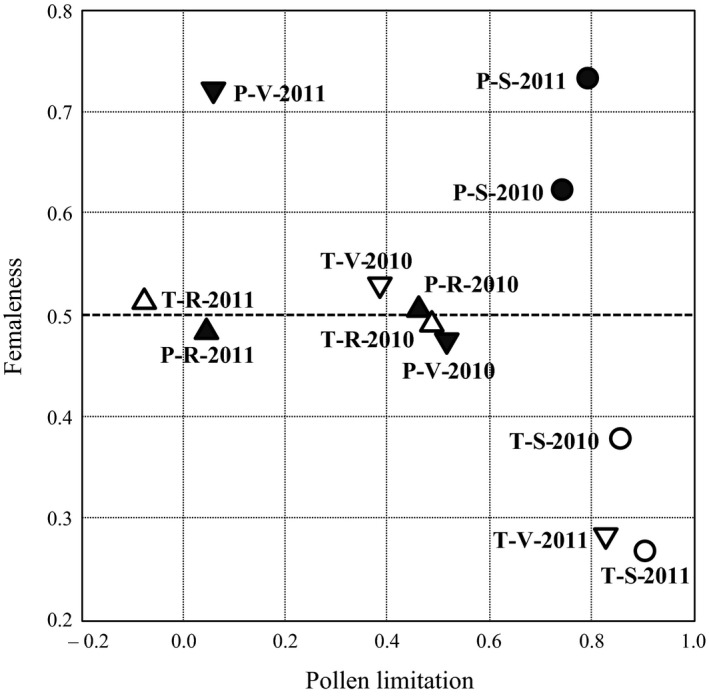
Relationship between pollen limitation and femaleness. Marks show the morphs (pin, closed; thrum, open) and sites (ridge, triangle; slope, circle; valley, inverted triangle). Text labels indicate the morph (pin, P; thrum, T), sites (ridge, R; slope, S; valley, V), and years (2010 and 2011).

The pollen limitation and femaleness of the plants are shown in Table 3d and e, respectively, and their relationships are illustrated in Figure 3. At the ridge, the pollen limitation was moderate and nonexistent during 2010 and 2011, respectively, irrespective of the morphs of the plant, and the femaleness of both morphs ranged from 0.48 to 0.52 over years. A sharp contrast was observed at the slope, where pollen limitation was extreme, and the femaleness of the pin and thrum plants ranged from 0.63 to 0.73 and 0.27 to 0.38, respectively. At the valley during 2010, the tendency was similar to that observed at the ridge, where pollen limitation was moderate and functional gender was not different between the morphs. At the valley during 2011, however, pollen limitation was nonexistent for the pin plants but extreme for the thrum plants, and the femaleness of the pin and thrum plants was 0.72 and 0.28, respectively.

### Dominant pollinator type

During the flowering period of *P*. *modesta*, the composition and visit frequencies of pollinators changed greatly (Table 5). Most pollinators of *P*. *modesta* were classified into either Diptera (e.g., Syrphidae and Anthomyiidae) or Hymenoptera (e.g., Halictidae, Andrenidae, Anthophoridae, and Apidae), and the visits by Lepidoptera (e.g., Papilionidae) and other insects were quite rare. The visit frequencies of Diptera simply increased as the season progressed. The visit frequencies of Hymenoptera, however, were highest at the ridge, lowest at the slope, and intermediate at the valley.

**Table 5:** Pollinators of *Primula modesta* observed within 2* *×* *2* *m plots at each site (ridge, slope, and valley) during 2011. Peak flowering time, flower density, observation time of pollinators, and visit number and visit frequency of pollinators are shown. Flower density (/m^2^) and visit frequency of pollinators (/flower/hour) are shown as mean per day* *±* *1 standard deviation

	Ridge	Slope	Valley
Peak flowering time	Early June	Early July	Late July
Flower density (/m^2^)	94.8 ± 25.1	98.8 ± 26.8	79.3 ± 13.0
Observation time of pollinators	25 h/5 days	12 h/3 days	14 h/4 days
Visit number of pollinators (number of flower visited in total)			
Diptera	211	272	385
Hymenoptera	303	25	62
Lepidoptera	9	12	8
Others	4	4	1
Total	527	313	456
Visit frequency of pollinators (/flower/hour)			
Diptera	0.0209 ± 0.0155	0.0531 ± 0.0391	0.0903 ± 0.0301
Hymenoptera	0.0331 ± 0.0195	0.0057 ± 0.0049	0.0157 ± 0.0042
Lepidoptera	0.0010 ± 0.0016	0.0023 ± 0.0012	0.0015 ± 0.0020
Others	0.0004 ± 0.0004	0.0011 ± 0.0015	0.0004 ± 0.0010
Total	0.0554 ± 0.0282	0.0622 ± 0.0414	0.1079 ± 0.0286

The accurate identification of pollinators was difficult to conduct in the field, and thus, we could not estimate the visit and pollination efficiencies of each pollinator. However, based on the field observations in accordance with the collection and identification of major pollinators, the most important legitimate pollinator of *P*. *modesta* was considered to be a long‐tongued Hymenoptera, *Eucera* sp. (Anthophoridae). It should be noted that we frequently observed this species visiting the flowers of *P*. *modesta* at the ridge; however, this species disappeared from the study area after early June, resulting in no visit by pollinators to the flower of *P*. *modesta* growing at the slope and valley. The long‐tongued bumblebees (Hymenoptera), such as *Bombus hypocrita* Pérez and *Bombus diversus* Smith (Apidae), were also considered to be legitimate pollinators; however, they rarely visited the flowers of *P*. *modesta*: The total number of flowers visited by bumblebees was only nine and once at the ridge and slope, respectively. Other long‐tongued pollinators present were Lepidopterans, such as *Papilio machaon* Linnaeus (Papilionidae); however, they rarely and sporadically visited the flower of *P. modesta*. All other pollinators collected were small, short‐tongued insects, which appeared to be illegitimate pollinators of *P. modesta*.

## Discussion

### Reproductive success of *Primula modesta*

In an alpine ecosystem, plants growing at the late snowmelt sites often suffer from resource limitation; a shortage of photosynthetic carbon gain due to a very short growth period results in a decrease in reproductive output (Kudo 1991; Berdanier and Klein 2011; Mallik et al. 2011; also see Leffler and Welker 2013). In the current study, the number of flowers per plant, ovules per fruit, and seed to ovule ratio by experimental crosses was smaller at the later snowmelt sites, apparently due to resource limitation. However, the effects of resource limitation on seed production did not differ between the morphs, and more importantly, the number of seeds produced under natural conditions was generally smaller than that produced by experimental crosses. This means that the pin and thrum plants of *P. modesta* growing at the same site have an equal ability of producing seeds if sufficient quantities of legitimate pollen grains are deposited on the stigma surface, but their seed‐set success was restricted by pollen limitation.

The strength of pollen limitation and functional gender (femaleness) of *P. modesta* varied greatly depending on the morphs and their growing sites, apparently because of the differences in pollination situations. At the ridge, where the flowers of *P. modesta* were frequently visited by a long‐tongued *Eucera* sp. (Hymenoptera), the femaleness of the pin and thrum plants was equal and constant, with no yearly variation, while the extent of pollen limitation over the years varied from nonexistent to moderate. At the slope, where the visit frequencies of Hymenoptera were lowest and most pollinators were composed of short‐tongued insects, the femaleness of the pin plants was 1.7–2.7 times that of the thrum plants, and the extent of pollen limitation was severe, irrespective of the morphs. At the valley during 2011, where visit frequencies of pollinators were highest but mostly composed of short‐tongued insects, the pin plants were functionally biased to the female with no pollen limitation, and the thrum plants were functionally biased to the male with severe pollen limitation. At the valley during 2010, however, the femaleness was not different between the morphs, and the extent of pollen limitation was moderate: A similar tendency observed at the ridge. Unfortunately, pollinators were observed only in 2011 and there are no quantitative data regarding the contribution of each pollinator to the reproductive success of *P. modesta*, which make it difficult to consider the causes of yearly variations. However, it is apparent that the variations of functional gender and pollen limitation observed in the current study are not ascribe to resource limitation nor biased morph ratio but to the seasonal changes in pollination situations along the snowmelt gradient.

The composition and activity of pollinators and the extent of resource and pollen limitation to the reproductive success of *P. modesta* observed in the current study show a sharp contrast with those reported by Shimono and Washitani (2007). Shimono and Washitani (2007) examined *P. modesta* growing at two sites (an oligotrophic flat fen site at 2100* *m a.s.l. and a southwest‐facing grassy slope at 2000* *m a.s.l.) in the subalpine zone of Mt Asama (N36°24′12″, E138°31′34″) in central Japan, approximately 70* *km away from our study sites. The number of ovules per fruit was significantly smaller at the fen site than at the grassy site, probably because of a shortage of the photosynthetic carbon gain due to the 2‐ to 3*‐*week delay in snowmelt and/or nutrient limitation due to the oligotrophy of the fen site. However, annual seed production was relatively constant, with no sign of pollen limitation regardless of the morph and site. Shimono and Washitani (2007) concluded that long‐tongued pollinators, *Empis flavobasalis* (Empididae) and *Bombylius major* (Bombyliidae), contribute to the effective and symmetric disassortative pollination of *P. modesta* at these sites. Here, it is noteworthy that both of these pollinators did not visit the flower of *P. modesta* at our study sites, indicating that the composition and activity of pollinators and the extent of resource and pollen limitation of *P. modesta* varied greatly among the regions.

### Evolutionary implications

Beach and Bawa (1980), following Robertson (1892) and Baker (1958), proposed that a replacement of pollinator types from long‐ to short‐tongued pollinators may result in flowers of distylous plants remaining morphologically and physiologically long styled (pin) and short styled (thrum), but only the pin flowers setting fruit and the thrum flowers donating pollen, because short‐tongued pollinators result in unidirectional pollen transfer from long stamens to long styles. In other words, asymmetrical disassortative pollination via short‐tongued pollinators may result in the functional gender specialization of the pin and thrum plants to more female biased and male biased, respectively. This hypothesis has long been invoked to discuss the evolutionary pathway followed from heterostyly to dioecy in the context of ecology (Bawa 1980; Beach 1981; Muenchow and Grebus 1989; Barrett and Richards 1990; McCall 1996; Pailler and Thompson 1997; Pailler et al. 1998; Barrett and Shore 2008; Haller et al. 2014); however, surprisingly few studies have examined this process in the field.

One exception is a series of studies of the style‐dimorphic plant genus *Narcissus* (Pérez‐Barrales and Arroyo 2010; Santos‐Gally et al. 2013a,b; Simón‐Porcar et al. 2014). Style dimorphism is an immediate ancestral state to distyly (Charlesworth and Charlesworth 1979; Lloyd and Webb 1992; Graham and Barrett 2004), where two floral morphs with long and short styles exist; however, anthers are approximately at the same height. In *Narcissus papyraceus* Ker‐Gawler, long‐tongued insects promote disassortative pollen transfer, and a pollinator shift from long‐tongued to short‐tongued insects results in low reproductive success of short‐styled plants, which may be related to the disappearance of short‐styled plants at the edge of their geographical distribution (Pérez‐Barrales and Arroyo 2010; Santos‐Gally et al. 2013b; Simón‐Porcar et al. 2014). Another exception is the study of distylous primrose, *Primula secundiflora* Franchet (Zhu et al. 2010, 2015). In this species, legitimate bumblebees contribute to the disassortative pollination between the pin and thrum flowers, but nectar‐robbing bumblebees only pollinated thrum flowers, and pollen‐collecting syrphid flies further pollinate pin flowers. Zhu et al. (2010, 2015) suggested that the complementary roles of bumblebee nectar robbers and syrphid flies contribute to sustain heterostyly in *P. secundiflora*. The results obtained by the studies of *Narcissus* and *Primula* are essential to consider the hypothesis of loss of long‐tongued pollinators by Beach and Bawa's (1980); however, the species examined in the former is not heterostylous, and the results obtained in the latter are contrastive (but not opposed) to the hypothesis, and both studies consider the pollinator compositions in a geographical scale.

The current study is remarkable in that asymmetrical disassortative pollination of distylous plants occurs in a local scale, apparently reflecting the seasonal changes in pollination situations. The seasonality in alpine ecosystem is largely determined by the time of snowmelt and can be stable for an extended time (Stanton et al. 1994; Kudo and Suzuki 1999; Thomson 2010). However, the conditions for the evolution of dioecy from distyly via the loss of long‐tongued pollinators (Beach and Bawa 1980) are considered to be restrictive; the long‐tongued pollinator must be eliminated from the population, and the sterility mutations in each morph must be tightly linked to the heterostyly supergene to prevent the abortion of the female part of the pin and the male part of the thrum (Muenchow and Grebus 1989). Recent individual‐based simulations by Haller et al. (2014) suggest that maladaptive gene flow between populations may mitigate these restrictions. At the present time, however, it is fair to state that “ecologically” dioecious plants need not evolve into obligately dioecious plants (Muenchow and Grebus 1989).

More importantly, the current study demonstrated that asymmetrical disassortative pollination of distylous plants often (even if not always) associated with severe pollen limitation. It is widely accepted that under severe pollen limitation, the ability of selfing (e.g., the morphological changes to promote self‐pollination, the genetic breakdown of self‐incompatibility, and the evolution of autonomous autogamy) often evolves to assure reproductive success (Darwin 1876; Baker 1955; Kalisz and Vogler 2003; Kalisz et al. 2004; Knight et al. 2005; Morgan and Wilson 2005; Moeller 2006; Evans et al. 2011). The breakdown of heterostyly into homostyly in response to pollen limitation has been reported in many heterostylous plants (Barrett 1988, 1989; Washitani et al. 1994; Guggisberg et al. 2006; Sakai and Wright 2008; Haddadchi and Fatemi 2015), which were often associated with the colonization into ecologically or geographically marginal areas. The severe pollen limitation of *P. modesta* provides the breakdown of heterostyly into homostyly more plausibility than the evolution of dioecy. Apparently, an accumulation of the empirical data is required demonstrating how phenological synchrony between plants and pollinators is decided and to what degree this relationship is stable over years (Forrest and Thomson 2011; Kudo 2014), along with estimates of selection and gene flow in individual plants to consider the possibility of adaptation by time in alpine plants.

## Conflict of Interest

None declared.
